# Geographic variation within the military health system

**DOI:** 10.1186/s12913-017-2216-1

**Published:** 2017-04-13

**Authors:** Linda Kimsey, Samuel Olaiya, Chad Smith, Andrew Hoburg, Stuart R. Lipsitz, Tracey Koehlmoos, Louis L. Nguyen, Joel S. Weissman

**Affiliations:** 1 Jiann-Ping Hsu College of Public Health, Georgia Southern University, Statesboro, GA USA; 2 Uniformed Services University of the Health Sciences, Bethesda, MD USA; 3 Brigham and Women’s Hospital, Boston, MA USA

**Keywords:** Geographic variations, Small area analysis, Healthcare organizations and systems

## Abstract

**Background:**

This study seeks to quantify variation in healthcare utilization and per capita costs using system-defined geographic regions based on enrollee residence within the Military Health System (MHS).

**Methods:**

Data for fiscal years 2007 – 2010 were obtained from the Military Health System under a data sharing agreement with the Defense Health Agency (DHA). DHA manages all aspects of the Department of Defense Military Health System, including TRICARE. Adjusted rates were calculated for per capita costs and for two procedures with high interest to the MHS- back surgery and Cesarean sections for TRICARE Prime and Plus enrollees. Coefficients of variation (CoV) and interquartile ranges (IQR) were calculated and analyzed using residence catchment area as the geographic unit. Catchment areas anchored by a Military Treatment Facility (MTF) were compared to catchment areas not anchored by a MTF.

**Results:**

Variation, as measured by CoV, was 0.37 for back surgery and 0.13 for C-sections in FY 2010- comparable to rates documented in other healthcare systems. The 2010 CoV (and average cost) for per capita costs was 0.26 ($3,479.51). Procedure rates were generally lower and CoVs higher in regions anchored by a MTF compared with regions not anchored by a MTF, based on both system-wide comparisons and comparisons of neighboring areas.

**Conclusions:**

In spite of its centrally managed system and relatively healthy beneficiaries with very robust health benefits, the MHS is not immune to unexplained variation in utilization and cost of healthcare.

## Background

According to the National Academy of Medicine (NAM), two of the primary drivers of excess cost in the U.S. healthcare system are the provision of unnecessary services and inefficiently delivered services [1]. If a health system wishes to remedy these issues, the science of performance improvement would suggest that reducing unwarranted variation in utilization and cost is the first step. However, variation, as it pertains to healthcare, can be both multifaceted and nebulous. Interest in variation of healthcare utilization can be traced to Lewis’ [2] analysis of tonsillectomy rates. Since then, numerous studies have examined geographic variation in different systems and populations – Medicare, the Veterans Health Administration, and privately insured patients - as it pertains to the delivery of healthcare services in the U.S. [3–6] and have consistently suggested that unexplained variation is too high. Research into the seemingly unexplainable differences in per capita cost between two Texas border towns [5] brought this issue to the public’s attention, although more recent work by Gawande suggests that costs have decreased in both towns since his earlier article [7]. Perhaps the most notable body of work on healthcare variation comes from the Dartmouth Atlas Working Group, focusing on variations in utilization and distribution of medical resources within the U.S. Medicare population [8–10].

Having been deemed “America’s undiscovered laboratory for health research” [11], the Military Health System (MHS) provides a unique window into healthcare geographic variation because it is a centrally controlled system using administered prices, providing healthcare to a captive population, at either very little or no out-of-pocket cost to enrollees. One might expect that utilization within an integrated system by universally insured beneficiaries to exhibit reduced variation. Yet, analysis performed by the Center for Naval Analysis did not find this to be the case [12, 13]. These researchers found large variation in utilization for joint replacements, Cesarean sections (C-sections), and coronary artery bypass grafting and percutaneous procedures for ischemic heart disease. However, variation was examined with facility as the unit of analysis and a focus on military facility costs, omitting utilization provided in the purchased care (civilian) sector. Bickett et al. [13] analyzed additional procedures and examined variation in cost of care for Navy beneficiaries living more than forty miles away from any Military Treatment Facility (MTF). Furthermore, these two studies examined only Navy facilities and/or Navy beneficiaries, omitting care provided to Army and Air Force beneficiaries who represent about 68% of the MHS total eligible beneficiaries [14].

In order to expand this area of inquiry, this study quantified geographic variation in utilization of back surgeries and C-Sections, two procedures of high interest to the MHS, and in per capita costs, across all system-defined regions within the entire MHS. Additionally, given differences in pricing/costing between military care and civilian care, the study used stratified analyses to examine whether systematic differences between its fee-for-service and budget-based components might play a part in variation. This study adds to the literature by analyzing geographic variation of a single centrally managed healthcare system containing both budget-based and fee-for-service components, which should provide valuable information for the MHS to inform future research and policy actions.

This project is a part of the Comparative Effectiveness and Provider Induced Demand Collaboration (EPIC), a joint effort of the Uniformed Services University of Health Sciences (USUHS) and Brigham and Women’s Hospital (BWH). The TRICARE data used for this study were obtained from the Military Health System Data Repository (MDR) under a data sharing agreement with the Defense Health Agency (DHA). TRICARE is the healthcare benefit program for the MHS, covering military active duty members, retirees, and family members. TRICARE is distinct from the health services provided by the Veterans Health Administration in that it serves those currently on active duty or in the Guard/Reserve, their families, and those who retire from a full military career. About 14.6% of beneficiaries are active duty service members. The remaining beneficiaries are spouses, children, and retirees [14]. Active duty members are automatically enrolled in Prime, a Health Maintenance Organization-like option (HMO), while retirees and dependents generally have an option between Prime, a Preferred Provider Option (PPO), and a traditional indemnity option. In some markets, TRICARE Plus offers Prime benefits to retirees over 65. TRICARE Prime and Plus beneficiaries, analyzed here, can be seen in either military facilities or in civilian facilities, depending upon availability within the region. The TRICARE population is socio-demographically comparable to the privately insured population.

TRICARE beneficiaries are assigned to a catchment area based on their zip code of residence. A MTF-based catchment area consists of zip codes within a 40-mile radius healthcare market area surrounding the MTF. Non-MTF (geography-based) catchment areas are comprised of zip codes that do not fall within 40 miles of a MTF, aggregated at the state (or sub-state) level [14]. This analysis studied ~3.4 million adult TRICARE Prime and Plus Enrollees for fiscal years 2007 through 2010 who were both living in a U.S. catchment area and enrolled to a primary care manager practicing in the U.S.

## Methods

In this study, the unit of analysis was the catchment area based on enrollee residence, of which there were approximately 110, depending on the year. Of these, approximately 47 were MTF-based, depending on the year. The remaining catchments were geography-based. Within each catchment, Prime enrollees could receive care from a MTF or from community-based civilian facilities, depending to some extent on both availability within the local military system and personal preference. Using fiscal year 2007–2010 data from the MDR, we evaluated per capita costs and utilization of two specific procedures that are common within the MHS: back surgery and C-section at the catchment level. Per capita costs included costs of inpatient and outpatient care in military and civilian facilities, as well as pharmacy, divided by the number of Prime enrollees.

Because back surgery has been regularly studied by the Dartmouth Atlas of Health Care, its widely-accepted definition was used to create this utilization measure [10]. The methodology of this research was modeled after that of the Dartmouth Atlas, in that the inpatient back surgery denominator included all enrollees identified for this study set (as opposed to the over-65 Medicare population used by Dartmouth Atlas researchers) [10]. The inpatient back surgery numerator was calculated using the same range of ICD-9-CM procedure codes, inclusive of a range of diagnosis codes, and excluding specific surgical codes, further modeling Dartmouth's methodology [10]. Rates of C-section as a percentage of total childbirths were analyzed because obstetrics is the largest service line in the MHS (verified by MDR analysis), and because of large variation across U.S. counties previously documented by Baicker, Buckles, and Chandra [15]. C-section rates were determined based on the count of C-sections represented by Medicare Severity-Diagnosis Related Groups (MS-DRGs) 765-768 and 774-775 as the numerator and the count of all births as the denominator, following Jaditz et al. [12]. Adjusted rates were then calculated, following Dartmouth Atlas methodology – adjusting for age, gender, and race [10]. Because our dataset contains race variables that are well-populated for active duty members (sponsors) but less so for their dependents, we imputed missing dependent race based on race of the sponsor, following Stewart et al. [16]. Coefficients of variation (CoV) and interquartile ranges (IQR) were then calculated for each measure for each year, using catchment based on enrollee residence as the geographic unit.

To investigate potential supply-side differences between MTF-based and non-MTF-based care, catchment areas were stratified into MTF-based and geography-based catchment areas, and annual CoV and IQR for each measure were calculated for each category. Stratification based on TRICARE’s three managed care support contract areas - North, West, and South regions - was also performed to investigate possible systematic differences due to contract management. Correlation of the relative percentage of patients receiving care in MTFs within a given catchment area to total (MTF plus non-MTF civilian care) utilization in the area was also examined using Pearson Product-Moment Correlation Coefficients to search for patterns that might indicate if greater (or lesser) care in military facilities were associated with greater (or lesser) utilization overall.

Finally, comparisons of MTF-based catchment areas to neighboring geography-based catchment areas were made to investigate the extent of differences in utilization. For example, Ireland Army Community Hospital – Fort Knox MTF catchment and the surrounding Kentucky geographic catchment were compared to each other. This was repeated for each of the MTF/geography-based catchment area pairs (48 in FY07; 44 in FY10) across the study period. Finding similar utilization patterns would suggest that geographic variation in the Military Health System is influenced to a greater extent by the geographic area in which facilities operate, as opposed to the military system of facilities to which it belongs. Using the adjusted incidence rates that were calculated for each catchment area, two-tailed hypothesis testing for differences in adjusted incidence rates for back surgeries and for differences in proportions for C-sections were performed. The Benjamini-Hochberg method [17] of false discovery rate correction was applied to the resulting *p*-values to adjust for multiple comparisons.

## Results

The average enrollee age across all catchments for FY 2010 was 29.6, reflecting the relatively young active duty population and their families. Males comprised 55.2% of the population. After assigning sponsor race for the race of dependents when missing (13.7%), and then removing records where this assignment was not possible (e.g., 4.6% were missing race of sponsor), 68.9% of the population was White, 18.0% of the population was Black, and the remaining 7.4% was American Indian/Alaska Native, Asian/Pacific Islander, or Other (Table 1). The remaining sample ranged from 3.2 million total covered lives in FY07 to 3.4 million in FY10. These statistics remained fairly consistent across the period of study. Catchment size varied widely, with a median of 25,353 (standard deviation (sd) 30,681) in FY 2010. Of note, the remaining findings were also analyzed limiting catchment size to at least 20,000 without significant changes in results.

**Table 1:** Sample catchment demographics

	FY07 (*N* = 110)	FY10 (*N* = 106)
Average Age (Std. Dev)	29.4 (3.41)	29.6 (3.24)
% Female	45.3%	45.2%
Race		
White	72.4%	68.9%
Black	18.0%	18.0%
Asian/Pacific Islander	4.3%	7.4%
American Indian/Alaska Native	1.4%	1.4%
Other	3.9%	4.3%
Catchment Size		
Quartile 1	13,365	12,915
Median	23,664	25,354
Quartile 3	38,853	44,720

**Fig. 1:**
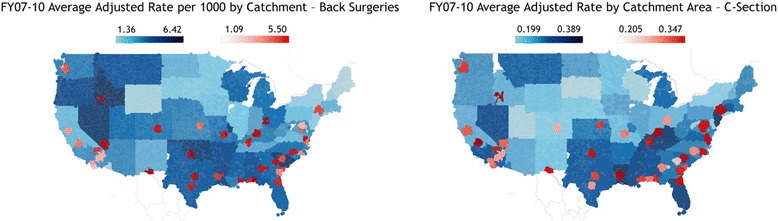
MHS catchment-level utilization maps: maps of catchment-level average adjusted rates for back surgeries and C-sections for FYs 2007 – 2010 [20]

**Fig. 2:**
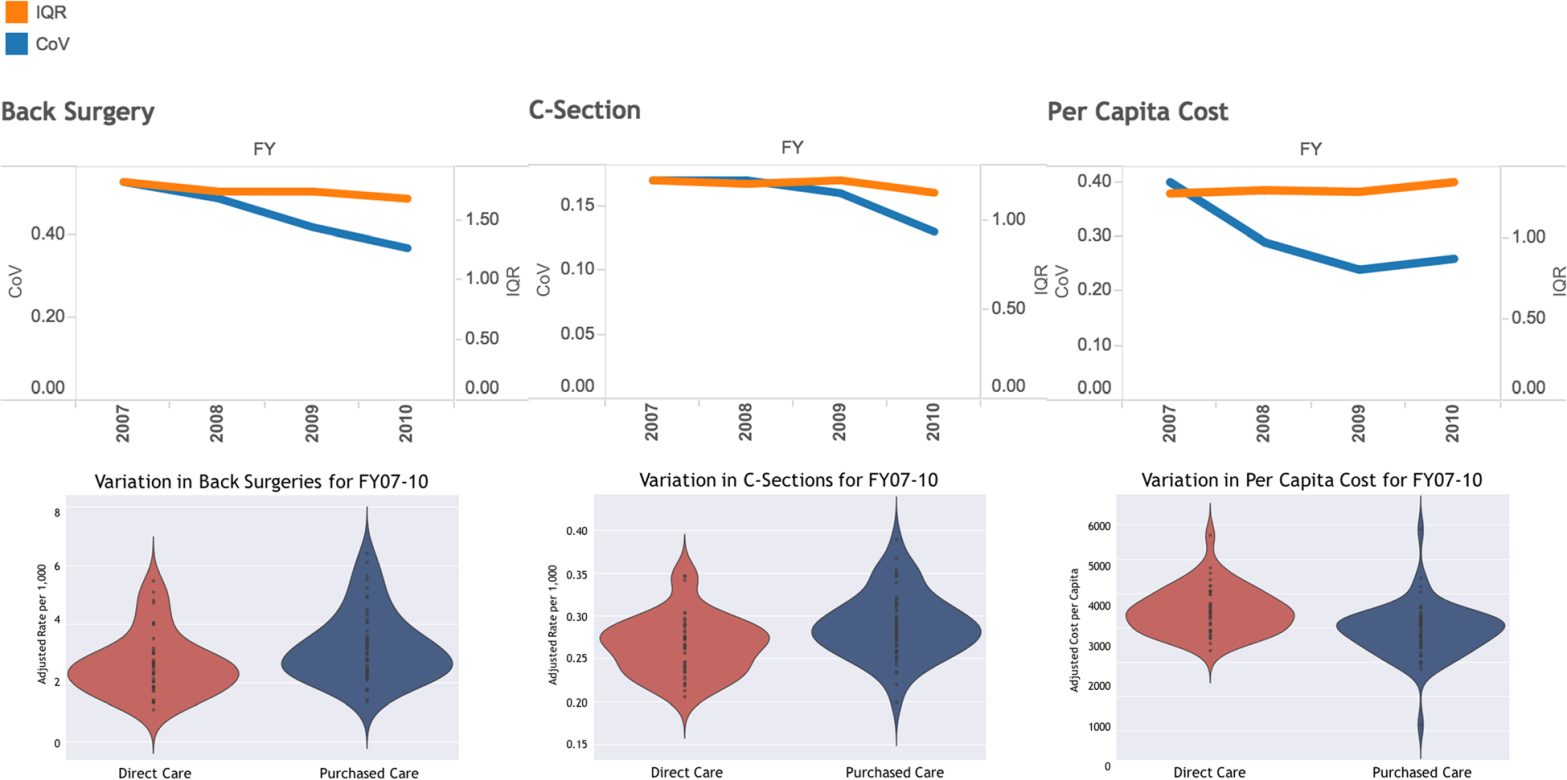
MHS catchment-level variation in utilization and per capita costs: catchment-level coefficients of variation, interquartile ranges (IQR), and violin plots of adjusted rates per thousand for back surgeries, C-sections, and per capita cost for FYs 2007 – 2010

Maps of catchment-level rates for back surgeries and C-sections are presented in Fig. 1. The maps illustrate how the catchments are defined: direct care catchments, in red, are focused near military facilities; geographic catchments, in blue, reflect areas where enrollees are eligible for care but are not near a military facility. Catchment-level coefficients of variation, interquartile ranges (IQR), and violin plots of adjusted rates per thousand are presented in Fig. 2. CoVs for 2010 were 0.37 for back surgeries (per 1,000) and 0.13 for C-sections (% of live births). 2010 IQRs were 1.7 and 1.2, respectively. The 2010 CoV (and average cost) for per capita costs was 0.26 ($3,479.51), with an IQR of 1.35. CoVs exhibited a slight decreasing trend over time for all measures with the exception of per capita costs in geography-based catchments.

**Fig. 3:**
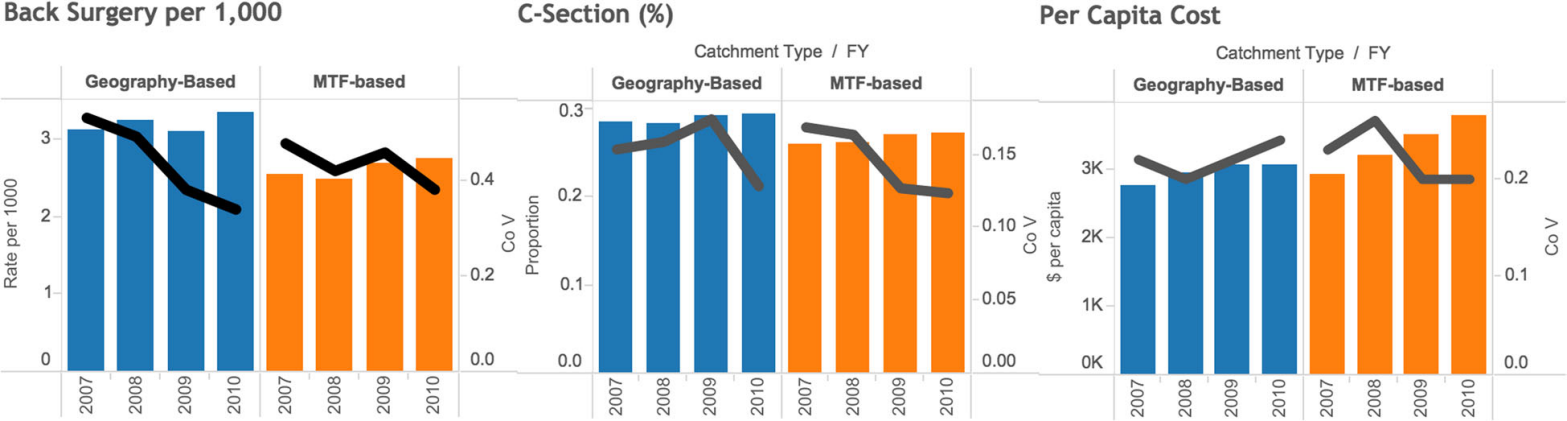
Utilization and coefficient of variation comparisons between MTF and geography-based catchments: back surgery utilization, C-section utilization, and per capita cost by catchment type for FYs 2007 – 2010

Comparisons of MTF-based catchments and geography-based catchments were performed to assess the possible contribution of systematic differences between the two to our results. This comparison revealed generally lower rates of back surgery (2010: 2.76 (+/- 0.32) vs. 3.35 (+/- 0.63); p = 0.093) and C-section (2010: 0.272 (+/- 0.01) vs 0.294 (+/- 0.01); p = 0.002) procedures in MTF-based catchments but similar CoVs between MTF-based and geography-based catchments. Statistical significance at a *p* < 0.05 level was attained each year for C-section, but only in 2007 for back surgeries. Total per capita costs, however, were higher in MTF-based catchments (2010: $3,923 (+/- $217) vs $2,970 (+/-$178)), and statistical significance at a *p* < 0.001 level was achieved in 2009 and 2010 (Fig. 3). Secondary analysis by catchment type revealed a consistently inverse correlation between the proportion of utilization that occurred in a military facility to total utilization for both back surgeries (2010: -0.31; 95% C.I. [-0.472, -0.127]) and C-sections (2010: -0.21; 95% C.I. [-0.312, -0.18]), meaning that catchment areas with a greater proportion of MTF-provided procedures tended to exhibit lower rates of overall utilization. Some regional patterns were noted. The West region exhibited moderate inverse correlation (2010: -0.53; 95% C.I. [-0.263, -0.723]) between overall catchment procedure rates and proportion of MTF-provided C-sections, while the South exhibited a similar inverse correlation (FY10: -0.56; 95% C.I. [-0.239, -0.773]) for MTF-provided back surgeries.

Comparisons of MTF catchments to their neighboring geographic catchment revealed similarities. Two-tailed hypothesis testing for differences in adjusted incidence rates for back surgeries found that for 56% of the catchment pair comparisons, there was no statistically significant difference (*p* < 0.05). In 42% of the catchment pair comparisons, rates were significantly lower for MTF-focused catchments; in the final 2% of the catchment pair comparisons, rates were significantly higher for MTF-focused catchments. Two-tailed hypothesis testing for differences in proportions of C-sections between pairs of MTF-based and neighboring geography-based catchments found that for 72% of the catchment pair comparisons, there was no statistically significant difference (*p* < 0.05). In 24% of the catchment pair comparisons, rates were significantly lower for MTF-focused catchments; in the final 4% of the catchment pair comparisons, rates were significantly higher for MTF-focused catchments (Table 2). These results suggest that the care provided to Prime beneficiaries in direct and purchased care settings is somewhat comparable.

**Table 2:** Tests for significance of differences in rates between MTF and geography-based catchments

Catchment counts	*MTF < Geography	No Statistical difference	*MTF > Geography
Back Surgery			
FY07	18	29	1
FY08	20	27	0
FY09	21	21	2
FY10	17	26	1
Average (% of Total)	41.5%	56.3%	2.2%
C-Section			
FY07	12	34	2
FY08	11	32	3
FY09	14	29	1
FY10	7	35	1
Average (% of Total)	24.3%	71.8%	3.9%

*p < .05

## Discussion

The MHS has several characteristics that suggest variation might be lower in the MHS than in the civilian sector. First, the entire MHS is centrally managed. Additionally, military medical personnel rotate among direct care facilities, providing an opportunity for knowledge and skill transfer across the system. Finally, all TRICARE Prime beneficiaries have a very robust health benefit, paying either zero or minimal cost shares. Expectations of low variation, however, were not supported in this analysis. Variation was generally high for the procedures examined. Our findings are in line with those of Jaditz et al. [12] and Bickett et al. [13] for Navy facilities and beneficiaries, discussed previously. Our findings are also in line with those of researchers examining civilian care. The CoV for Medicare back surgeries, based on Dartmouth Atlas Health Referral Regions, was 0.29 in 2010 [6], and Weinstein et al. found procedure-specific CoVs of 0.346 for lumbar discectomy/laminectomy and 0.495 for lumbar fusion [18]. With respect to C-sections, Epstein and Nicholson documented CoVs for 2003 C-section rates at 0.12 for Florida and 0.13 for New York at the health district level [19].

Stratification by MTF-based and geography-based catchment areas revealed generally lower utilization and higher per capita costs for MTF-based areas. However, neither correlation of total catchment utilization to percentages of direct care provided within a catchment area, nor comparisons of MTF-based catchments to neighboring geography-based catchments indicated systemic differences between the two. The disconnect between utilization and costs across the direct and purchased care catchment areas is an area worthy of further analysis. In addition, focusing future research on the subpopulation level (i.e., family members or retirees) to control for possible patient-level variation due to unique healthcare needs of injured service members may be informative.

Our analysis was subject to several limitations. Catchments were determined based on patient residence, as is the case with the Dartmouth Atlas [10]. However, in the MHS, residence and treatment catchments may differ, especially for care rendered in the direct sector: beneficiaries may receive care, especially surgeries, at a MTF even though they live outside of the MTF catchment area. Differences in accounting methods between the direct care sector (patient-level cost allocation of budgeted funds) and the purchased care sector (claim reimbursement basis) complicate direct comparisons of costs, and may contribute to an explanation of why per capita costs were higher in MTF-based catchments even though utilization was lower. Problems with missing race variables for dependents required a proxy of sponsor race for imputation. Finally, our analysis followed Dartmouth Atlas methodology, meaning that risk adjustment for patient health status was not performed. Health status has been found to account for a significant amount of variation in healthcare [6], so not adjusting for it could mean our analysis has overstated the amount of variation present.

## Conclusions

Variation is thought to be an indicator of possible inefficient and/or ineffective care. While the unsystematic U.S. healthcare system might be expected to exhibit a fair amount of variation, the centrally managed system of the MHS, with its universally insured population, is an environment where variation might be minimized. However, this study found significant variation within the MHS, comparable to studies of other populations, for the measures analyzed. Further work to understand this variation could help to shape future managed care support contracts and direct care practices, perhaps by the inclusion of well-designed incentives to minimize variation through the use of clinical guidelines. Because it is a system, efforts to reduce variation may have a greater chance of success.

## Abbreviations

BWH: Brigham and women’s hospital
CoV: Coefficients of variation
DHA: Defense health agency
EPIC: Comparative effectiveness and provider induced demand collaboration
HMO: Health maintenance organization
IQR: Interquartile range
MDR: Military health system data repository
MHS: Military health system
MS-DRG: Medicare Severity-Diagnosis Related Group
MTF: Military treatment facility
NAM: National academy of medicine
PPO: Preferred provider option
SD: Standard deviation
USUHS: Uniformed services university of health sciences
